# Intraspecific genetic lineages of a marine mussel show behavioural divergence and spatial segregation over a tropical/subtropical biogeographic transition

**DOI:** 10.1186/s12862-015-0366-5

**Published:** 2015-05-31

**Authors:** Gerardo I Zardi, Katy R Nicastro, Christopher D McQuaid, Rita Castilho, Joana Costa, Ester A Serrão, Gareth A Pearson

**Affiliations:** Department of Zoology and Entomology, Rhodes University, Grahamstown, 6140 South Africa; Centre of Marine Sciences – CCMAR, Campus de Gambelas, Universidade do Algarve, 8005-139 Faro, Portugal; Present address: Algal Biology Lab, School of Botany, The University of Melbourne, Parkville, 3010 VIC Australia

**Keywords:** Genetic diversity, Marine connectivity, *Perna perna*

## Abstract

**Background:**

Intraspecific variability is seen as a central component of biodiversity. We investigated genetic differentiation, contemporary patterns of demographic connectivity and intraspecific variation of adaptive behavioural traits in two lineages of an intertidal mussel (*Perna perna*) across a tropical/subtropical biogeographic transition.

**Results:**

Microsatellite analyses revealed clear genetic differentiation between western (temperate) and eastern (subtropical/tropical) populations, confirming divergence previously detected with mitochondrial (COI) and nuclear (ITS) markers.

Gene flow between regions was predominantly east-to-west and was only moderate, with higher heterozygote deficiency where the two lineages co-occur. This can be explained by differential selection and/or oceanographic dynamics acting as a barrier to larval dispersal.

Common garden experiments showed that gaping (periodic closure and opening of the shell) and attachment to the substratum differed significantly between the two lineages. Western individuals gaped more and attached less strongly to the substratum than eastern ones.

**Conclusions:**

These behavioural differences are consistent with the geographic and intertidal distributions of each lineage along sharp environmental clines, indicating their strong adaptive significance. We highlight the functional role of diversity below the species level in evolutionary trends and the need to understand this when predicting biodiversity responses to environmental change.

**Electronic supplementary material:**

The online version of this article (doi:10.1186/s12862-015-0366-5) contains supplementary material, which is available to authorized users.

## Background

The early recognition of functionally significant levels of biodiversity within a species is a central objective of evolutionary biology and biodiversity conservation [1]. This is particularly important at times when biodiversity loss is one of the planet´s major global problems [2,3]. Biodiversity can be investigated, and its importance evaluated, at different levels including species, their genes and functional traits [4,5].

Given the recurrent detection of distinct phylogeographic lineages, it is clearly necessary to consider the adaptive significance of intraspecific differences in order to understand the evolutionary potential of a species [6,7]. Complementary approaches based on neutral genetic markers, commonly employed to estimate recent demographic connectivity, and on methods that can reveal ecologically relevant adaptive traits across heterogeneous habitats are pivotal for the assessment and management of intraspecific diversity (e.g. [8,9]).

The identification of evolutionarily significant units uniquely based on neutral molecular markers, while overlooking intraspecific patterns of phenotypic divergence, can lead to underrepresentation of locally adapted populations and thus underestimate biodiversity [10,11]. Local environmental conditions resulting in spatially divergent selective regimes can maintain local adaptation across populations of the same species independently of varying and/or contrasting neutral genetic structure [12,13].

Species with wide distributions over variable environments and large effective population sizes often display marked intraspecific biodiversity with distinct genotypes/populations within a species, resulting from adaptation to local environmental conditions but capable of interbreeding with other ecotypes of the same species (e.g. [13,14]). Marine broadcast spawners with broad geographic ranges extending over abrupt environmental clines are ideal model organisms for studying the interplay between local adaptation and genetic structure due to neutral processes, because divergent selection produced by distinct environments is coupled with a high potential for gene flow among geographically separated subpopulations through larval transport.

**Figure 1:**
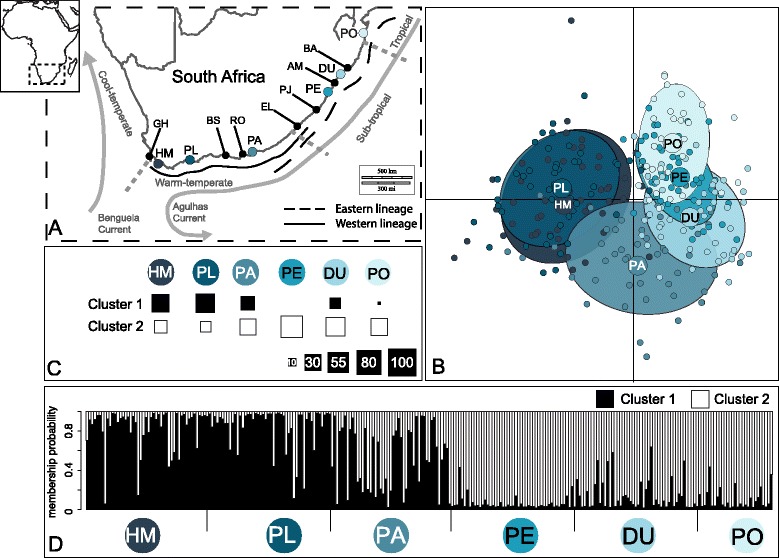
Study area and genetic results. **A)** Map of the study area and sampling sites. Black dots: localities either mentioned in the text or used for ecologic data; blue dots: localities used for genetic data abbreviated as in Table 1. Continuous, black line: distribution of the western lineage (according to [18]); dashed, black line: distribution of the eastern lineage (according to [18]). The three biogeographic regions of southern Africa (according to [77]) are delimited by grey dotted lines. **B)** Results of the Bayesian approach using the Discriminant Analysis of Principal Components (DAPC) scatter plot of individuals with *a priori*-defined locations, to investigate genetic structure. Labels were placed at the centre of dispersion of each group, which are delineated by inertia ellipses. **C)** DAPC *a posteriori* genetic cluster assignment for K = 2. The size of each square is proportional to the percentage of individuals assigned to each cluster at each location. **D)** Stacked bar graph of assignment probabilities per individual within *P. perna* locations across the southern tip of South Africa. Assignment proportions for each individual were obtained from Structure, based on genotypes from seven nuclear microsatellite loci, for K = 2.

The southern African coastline covers a wide range of climatic and oceanic conditions and can be divided into three major biogeographic regions: cool-temperate (from southern Namibia to the Cape of Good Hope; Figure 1A), warm-temperate (from the Cape of Good Hope eastward to East London) and sub-tropical (from East London north to Mozambique). The environmental discontinuities characteristic of these different parts of the coastline are mirrored by a degree of endemism among the different biogeographic provinces and phylogeographic breaks in species that occur in more than one province ([15] and references therein, [16]).

The brown mussel *Perna perna* is a habitat-forming species distributed widely around the world in warm waters. In southern Africa, it dominates intertidal shores in the sub-tropical and warm-temperate bioregions (from central Mozambique to the Cape of Good Hope, GH). It is absent from there to central Namibia due to the cold, upwelled waters of the Benguela system (a distributional gap of more than 1000 km; Figure 1A). It then extends northwards along the west coast of Africa to the southern Iberian Peninsula and into the Mediterranean Sea as far as the Gulf of Tunis ([17] and references therein).

Analyses of mitochondrial DNA (mtDNA) sequences indicate a sharp phylogeographic break on the southeast coast of South Africa (corresponding to the warm-temperate/subtropical transition) forming a western and an eastern lineage [18]. The western lineage includes animals from both the Namibian coast and the south coast of South Africa (despite the wide gap across the Benguela upwelling system). The eastern lineage includes individuals from the southeast and east coasts of South Africa. The two lineages overlap in their distributions on the southeast coast over a distance of approximately 200 km. The presence of the two lineages was recently confirmed by nuclear (ITS) sequence data [19]. Most importantly, mitochondrial (COI) and nuclear (ITS) markers clearly show a non-sister relationship for the two South African lineages [19]. The most likely biogeographic scenario explaining the independent origins of the two South African lineages involves an Indo-Pacific origin for *P. perna*, dispersal into the Mediterranean and Atlantic through the Tethys seaway, and recent secondary contact after southward expansion of the western and eastern South African lineages [19]. Manipulative experiments and the release of oceanographic drifters suggest that phylogeographic patterns of *P. perna* may be maintained by a combination of local conditions experienced in the different biogeographic regions, and by the isolating effect of regional oceanographic dynamics [20].

Here, we examine the hypotheses that contemporary demographic connectivity and functional traits of *P. perna* populations are influenced by the tropical/subtropical biogeographic transition. First, we adopted a population genetic approach using microsatellite markers, which are unaffected by natural selection, to investigate differentiation and migration patterns of *P. perna* between populations inhabiting different bioregions. The re-examination of genetic structure in South African *P. perna* with microsatellites and comparison with previously reported mitochondrial data is critical to assess potential mito-nuclear discordance and contemporary biogeographic conditions. Second, we used a common-garden design to compare fundamental behavioural traits known to affect survival along large (tropical/subtropical) and small (within-shore) scale environmental gradients. In particular, we assessed shell gaping and attachment strength, key attributes affecting tolerance of the two major stresses in intertidal habitats, desiccation and wave action. A common-garden design is necessary to distinguish between adaptive and plastic responses of population subjected to distinct selective pressures. Finally, we compared intertidal elevation (vertical distributional limits) of the two lineages by using *in situ* temperature profiles to compare intertidal limits of populations from the two bioregions.

## Materials and methods

### Ethics statement

Mussel collection were performed under permit number RES2014/12 issued by the Department of Agriculture Forestry and Fisheries to the Department of Zoology and Entomology at Rhodes University.

### Genetic data

#### DNA extraction, amplification and genotyping

Mussels (adults, >4cm in shell length) were collected between February 2010 and May 2013 at six locations in southern Africa (Figure 1A and Table 1) in the middle portion of the species’ vertical distribution. Mantle tissue (20-30 mg) was dissected from each individual, preserved in 96% ethanol and stored at -20°C. Total genomic DNA extraction was performed using a standard Proteinase K protocol adapted from [21]. Molecular genotyping of a total of 269 samples was carried out using a set of 10 microsatellite loci in three multiplex reactions, with subsequent separation of the PCR products using an ABI PRISM 3130xl DNA analyser (Applied Biosystems) with GeneScan Liz 500 as size standard (Applied Biosystems) and following the procedure of Coelho et al. [22]. Data were scored using PEAK-SCANNER v. 1.0 software (Applied Biosystems).

**Table 1:** **Genetic diversity of each population**

**Locations**	**Codes**	**Coordinates**	**N**	**H**_**E**_	**H**_**O**_	**F**_**IS**_	**Â (±SD)**
Hermanus	HM	−34.41, 19.26	48	0.811	0.714	0.130*	14.36 (±0.55)
Plettenberg Bay	PL	−34.00, 23.46	48	0.799	0.679	0.161*	14.2 (±0.16)
Port Alfred	PA	−33.48, 27.15	47	0.802	0.637	0.216*	14.74 (±0.29)
Port Edward	PE	−31.05, 30.23	48	0.796	0.740	0.084*	15.7 (±0.45)
Durban	DU	−29.55, 31.22	48	0.829	0.718	0.145*	16.3 (±0.17)
Ponta do Ouro	PO	−26.84, 32.89	30	0.777	0.759	0.045	15.6 (±0)

Locations are depicted in the map of Figure 1A and are ordered from west to east. Decimal coordinates (latitude and longitude, respectively); N: sample sizes; H_E_ and H_O_: expected and observed heterozygosity; F_IS_: inbreeding coefficient with significant values after correction for multiple test indicated with *(p < 0.05); Â (±SD): allelic richness (standardized for N = 30 by resampling) ± standard deviation.

#### Location-based analyses

Scoring errors, large allele dropout and null alleles were checked employing the program MICROCHECKER [23] and frequency of null alleles estimated based on the algorithm presented in Brookfield [24]. Additionally, tests for Hardy–Weinberg equilibrium were conducted using GENETIX 4.05 software [25] for all locus-location combinations by assessing the inbreeding estimator Wright’s F_IS_ and estimating significant values after 10,000 permutations. Tests for linkage disequilibrium between all pairs of loci were performed according to the method of Black and Kraftsur [26] implemented in GENETIX.

For each location, allele frequencies, observed heterozygosity (H_O_) and expected heterozygosity (H_E_), plus F_IS_ (significance based on 1,000 permutations and adjusted using q-value correction for multiple comparisons; [27]) were computed with GENETIX. Standardized allelic richness (Â) was estimated by resampling 1000 times to standardize to the smallest sample size (n = 30) to account for differences between sampled locations, using StandArich R package [28]. Pairwise location estimates of genetic differentiation plus 95% bootstrapped confidence intervals (10,000 replicates) were estimated using Weir and Cockerham’s [29] F_ST_ estimator (θ) and Jost’s [30] D_ST_. Calculations were executed in the Diversity R package [31].

#### Estimating admixture

To evaluate the extent of admixture between the two mitochondrial lineages using microsatellite data, we used STRUCTURE 2.2 [32]. Clusters (K) varied from one to a maximum of seven, corresponding to the number of locations in the study data plus one. Twenty replicate runs [100,000 Markov Chain Monte Carlo (MCMC) iterations and 10,000 burn-in**]** were performed under the admixture and the correlated allele frequency model. The most probable value of K was inferred based on the ad hoc criteria L(K) proposed by Pritchard et al. [32] and delta K by Evanno et al. [33]. Similar replicate runs were grouped based on the symmetric similarity coefficient of >0.9 using the *FullSeach* algorithm in CLUMPP 1.2 [34] and visualized with bar_plotter.rb 1.1 implemented in Ruby.

To complement the results of STRUCTURE, we used discriminant analysis of principal components (DAPC, [35]), which can be performed when uncovering population admixture in the absence of assumptions. This method first transforms the data using principal components analysis, which ensures that the variables are not correlated and that the number of variables is smaller than the number of individuals. We ran two DAPC analyses in ADEGENET R package [36], following Jonker et al. [37]: a location assignment and a genetic cluster assignment. We assigned each individual *a priori* to its location of origin and obtained for each one the probability of assignment to the correct sampling site. This procedure maximizes the among-location variation and minimizes the within-location variation [35]. Using the *find.clusters* function (with 10^7^ iterations), we ran successive K-means clustering of the individuals from K = 1 to K = 6, and identified the best supported number of clusters through comparison with the Bayesian Information Criterion (BIC) for the different values of K. We determined the number of clusters and assigned each individual to a genetic cluster without providing any *a priori* population assignment. In that way, the grouping factor is the genetic cluster and not the sampling location, offering an unbiased interpretation of population structure. To avoid unstable assignments of individuals to clusters, we retained 89 PCs (sample size divided by three), but used all discriminant functions, in a preliminary DAPC run. The results were then reiterated by the optim.a.score function with 100 simulations to determine the optimal number of PCs, and a final DAPC was subsequently carried out with the optimal number of PCs.

#### Estimating the directionality of gene flow

To compare different biogeographic hypotheses on migration rates of *P. perna*, we used the coalescent-based program Migrate-N MIGRATE version 3.1.3 [38,39]. This approach assumes the Wright-Fisher model, where locations have a constant effective size through time, the rate of mutation is constant, and locations exchange migrants with constant rates per generation, but those rates can vary among locations. We conducted the analyses on a random subsample of 30 individuals, with the dataset structured into three groups according to the genetic clusters recovered (i.e. all populations were assigned to one of the two clear clusters with the exception of PA, which fell indistinctly into each of the two, and so was considered separately): West (sites HM, PL), Centre (PA) and East (PE, DU, PO). We tested four variations of the three-group (West, Centre, East) migration model: all directional migration (Model 1: West ⟷ Centre ⟷ East, full model, nine parameters), strict west to east migration (Model 2: West → Centre → East, six parameters), strict east to west migration (Model 3: West ← Centre ← East, six parameters) and from the centre to the margins (Model 4: West ← Centre → East, five parameters). Testing the directionality of gene flow is justified because the dominant ocean current between the ocean basins, the Agulhas Current, runs westerly from the Indian towards the Atlantic Ocean (Model 3) and is thought to play a role in limiting marine dispersal in the opposite direction [40,41] though coastal counter-currents also occur [42] (Model 2). Models 1 and 4 consider indistinct migration among groups and migration directionality towards the transition area between the two clusters respectively. Initial values were calculated using *F*_ST_. Mutation rates were set to be constant among loci. The Migrate-N run parameters were calibrated on the full model for convergence of the Markov chain Monte Carlo sampling method. The prior distributions were uniform for mutation-scaled population size parameters theta (θ), that are four times the product of the effective population size and the mutation rate, and mutation-scaled migration rates M, that is, migration rate scaled by the mutation rate, over the range of θ = 0–100 and M = 0–100. These settings resulted in converged posterior distributions with a clear maximum for each estimate. The three replicate Bayesian runs consisted of one long chain with a total of 6 million states and genealogy changes after discarding the first 10,000 genealogies as “burn-in” for each locus. For all the analyses we used an adaptive heating scheme with 4 simultaneous chains using different acceptance ratios (temperature settings were 1.0; 1.5; 3.0; 1,000,000.0). Overall loci information was combined into a single estimate by Bézier approximation of the thermodynamic scores as described by Beerli and Palczewski [43] and we averaged the Bézier score over three different runs and used this as input to evaluate multiple models using Bayes factors [44].

### Ecological data

#### Attachment strength and gaping behaviour

All mussels used for the assessment of behavioural traits were collected from locations known to support pure mtDNA lineages ([18]). Specimens (shell length 3-4cm) were collected in February 2013 [at Brenton-on-Sea (BS) for the western lineage and at Amanzinoti (AM) for the eastern lineage; Figure 1A) and acclimated in seawater at 17°C for two weeks prior each experiment. A second trial was replicated in February 2015 with mussels from two additional locations [at Robberg (RO) for the western lineage and at Port St. Johns (PJ) for the eastern lineage; Figure 1A]. Common garden experiments involve the acclimation and comparison of individuals from distinct geographical locations under identical environmental conditions. Such experimental set-ups are widely used to disentangle the effects of genetic and environmental conditions on observed phenotype differences (e.g. [45]).

Mussels were divided in three separate tanks (n =5 of each lineage in each tank), where they were placed at least 10cm apart so that they maintained a solitary position (i.e. horizontal to the substratum) and attachment strengths were not influenced by nearby conspecifics. After three days, the attachment strength of each mussel was measured by drilling a hole into one of the valves and using a spring balance to record the force required to dislodge the mussel perpendicularly to the substratum [46].

Mussels (n = 15 for each lineage and each measuring interval) were exposed to air for 3h in a controlled environment chamber at 35°C and 60-80% humidity. For each one hour interval, the number of valve movements (gaping behaviour) were noted by visual observation, without recording the width of the gape [47].

#### Intertidal distributional limits

Temperature dataloggers (iButtons®, Maxim Integrated Products, Dallas Semiconductor, USA) were used to relate the effective shore height of *P. perna* at two open coast sites on the east coast [Amanzinoti (AM) and Balito (BA); Figure 1A] and two within the western range of the species (BS and RO)*.* At each site, two transects were established, approximately 50 m apart and running perpendicular to the shoreline. Along each transect, two logger were deployed at the upper and two at the lower limit of *P. perna* distribution. Loggers were programmed to record data at 10 min intervals between February 1^st^ to 14^th^ of 2013 (western range) and March 17 to 30 of 2014 (eastern range), covering the two-week tidal cycle in both regions (i.e. a neap and a spring tide). Temperature logger profiles clearly reveal when loggers are first inundated by the returning tide (sudden, sharp temperature drops; ≥ 3°C over 10 min; [48]). This was used to calculate the effective shore height using the online forecasts service of SHOM database (http://www.shom.fr; settings: hauteur d’eau à une heure donnée). Because tide cycles are not in phase across large geographical scales, Durban and Mossel Bay tidal elevations were used for the east and south coast respectively. Where both loggers were recovered at a transect, the values were averaged and used to estimate populations’ intertidal limits at each transect.

#### Data analyses

Each trial was analysed separately. Attachment strength data were analysed using a 2-way ANOVA with Lineage (western or eastern) and Block (tank 1, 2 or 3) as fixed and random factors respectively. Gaping behaviour data were analysed using a 2-way ANOVA with Lineage (western, eastern) and Time (hour 1, 2 or 3) as fixed factors.

For each intertidal Limit (low, high), data were analysed under a nested design with Range (west, east) as a fixed factor, Site (1, 2) nested within Range and Transect nested within Range and Site.

Heteroscedasticity was tested using Levene’s test and *post-hoc* separation of significant means by SNK tests. Analyses were performed in SPSS (IBM Corp., USA).

## Results

### Genetic data

Null alleles at high frequencies (>0.2) were indicated by MICROCHECKER for three loci (P11, P16, P27) for several localities (Additional file 1: Figure A1). For these three loci, departures from HWE were consistently reported across all localities. To avoid overestimation of F_IS_ and F_ST_ estimators [49] by null alleles, these three loci were removed from all subsequent analyses, including descriptive analyses reported in Table 1. Particularly high null allele frequencies have often been reported in several invertebrate taxa, including corals and bivalves, due to high levels of DNA sequence variation [50]. Specifically, nucleotide variations in microsatellite flanking regions can cause a high frequency of null-alleles, even within populations used for characterization of microsatellites [51].

All microsatellite loci were highly polymorphic among the six populations. The 269 individuals contained 226 alleles in these loci, with an average number of alleles per locus of 33, ranging from 9 (P29) to 98 (P20; Additional file 2: Figure A2). Allelic richness and gene diversity H_E_ varied between 14.2-16.3 and 0.829-0.777 respectively and the highest allelic richness was recorded at DU (Table 1). Mean heterozygote deficiency was detected at all locations with the exception of PO (Table 1 and Additional file 3: Table A1 for estimates for each locus).

Genetic differentiation among populations was detected as indicated by pairwise F_ST_ and D_ST_ values (p < 0.05 for all comparisons with the exception of HM *vs.* PL and PE *vs.* PO; Table 2). While D_ST_ pairwise differentiation was lower within than among two groups (hereafter ‘western’ group: HM, PL; and ‘eastern’ group: PE, DU, PO), F_ST_ values did not clearly differentiate PA as belonging to either group.

**Table 2:** **Genetic differentiation between pairs of populations**

**Location**	**HM**	**PL**	**PA**	**PE**	**DU**	**PO**
HM		0.002	**0.065**	**0.289**	**0.192**	**0.287**
		(-0.018, 0.032)	(0.024, 0.113)	(0.216, 0.377)	(0.142, 0.262)	(0.205, 0.381)
PL	0		**0.075**	**0.316**	**0.200**	**0.313**
	(-0.007, 0.008)		(0.029, 0.132)	(0.237, 0.404)	(0.142, 0.271)	(0.228, 0.413)
PA	**0.047**	**0.058**		**0.207**	**0.120**	**0.256**
	(0.03, 0.068)	(0.04, 0.081)		(0.142, 0.281)	(0.064, 0.187)	(0.157, 0.355)
PE	**0.077**	**0.088**	**0.047**		**0.069**	0.022
	(0.06, 0.1)	(0.071, 0.108)	(0.033, 0.065)		(0.021, 0.127)	(-0.018, 0.08)
DU	**0.072**	**0.082**	**0.031**	**0.021**		**0.053**
	(0.057, 0.09)	(0.067, 0.098)	(0.016, 0.048)	(0.01, 0.033)		(0.01, 0.118)
PO	**0.080**	**0.089**	**0.073**	0.009	**0.032**	
	(0.06,0.107)	(0.067, 0.114)	(0.048, 0.105)	(-0.007, 0.032)	(0.015, 0.053)	

Codes correspond to locations in Figure 1A and are ordered from west to east. Pairwise genetic differentiation (F_ST_, below the diagonal, D_ST_ above the diagonal). 95% confidence intervals are shown in brackets and confidence intervals not enclosing 0 are represented in bold.

Discriminant Analysis of Principal Components (DAPC), run with geographic location defined *a priori*, clearly differentiated the ‘western’ and ‘eastern’ groups on the scatter plot x-axis, confirming the presence of the genetic structure indicated by the *F*_ST_ analysis (Figure 1B). Individuals from PA contained an admixture of alleles from both groups and indistinctly clustered with each of them. When the *a posteriori* genetic cluster assignment was run for K = 2 (best supported number of clusters), most individuals of the ‘western’ group were assigned to the same cluster (cluster 1; Figure 1C). The individuals of the ‘eastern’ group were also mostly assigned to one cluster (cluster 2), and in a higher rate than the populations of the first cluster. This is particularly true for PE, where all individuals were assigned to cluster 2. Individuals from PA were assigned to both clusters with a slightly higher number of individuals assigned to cluster 2.

This pattern of subdivisions was largely consistent with the results obtained with STRUCTURE, which revealed the most significant increase of ΔK at two clusters, (Figure 1D), thereafter ΔK remained unchanged. The L(K) plot rejected the option of K = 1. We concluded that K = 2 is the most likely number of genetic clusters (Additional file 4: Figure A3). While at most locations individuals displayed an admixture higher than 0.85 for either group or cluster, PA showed a split of 0.53 and 0.47 for the western and eastern groups respectively.

When comparing the four biogeographic hypotheses with MIGRATE-n, Model III, strictly east to west migration, was the only model supported (Table 3).

**Table 3:** **Bayes factors model comparison of migration models for** ***P. perna***

**Models**		**Model parameters**			**Bézier**	**dBézier**	**Probability**
		**Set 1**	**Set 2**	**Set 3**			
Model 1	W↔ C ↔ E and W ↔ E	***	***	***	−763993	−321213	0
Model 2	W → C → E and W → E	*00	**0	***	−459610	−16830	0
Model 3	W ← C ← E and W ← E	***	0**	00*	−442780	0	1
Model 4	W ← C → E	**0	0*0	0**	−459610	−16830	0

Model parameters code as follows: the first set of three signs indicate estimation of theta for West, migration from Centre to West, migration from East to West; the second set indicate migration from West to Centre, estimation of theta for Centre, migration from East to Centre; the third set indicates migration from West to East, migration from Centre to East and estimation of theta for East. An asterisk indicates that that particular parameter was estimated by the model, and a 0 indicates that no migration was allowed.

### Ecological data

**Figure 2:**
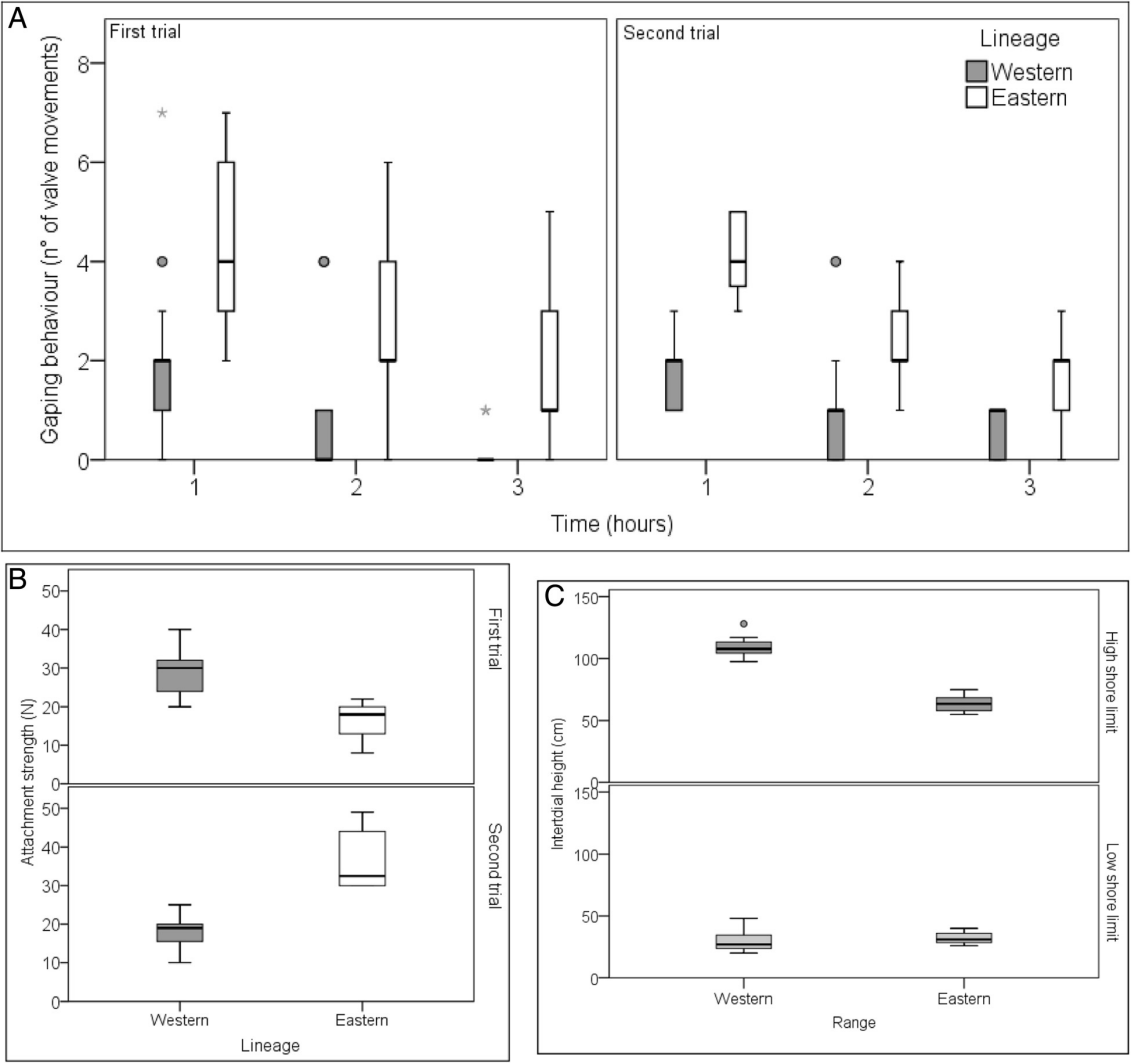
Ecological results. **A)** Gaping behaviour of the eastern and western lineages, each trial is plotted separately, **B)** Strength of attachment (blocks pooled), each trial plotted separately, **C)** High and low intertidal limits of *P. perna* western and eastern lineages. Dots depict mild outliers (i.e. outliers outside the inner fences) and stars are extreme outliers (i.e. outliers outside the outer fences).

In each trial, lineage (as defined by mtDNA in [18]) was a highly significant factor influencing attachment strength (two-way ANOVA, square-root transformed, p < 0.01; Figure 2A), with the eastern lineage having a higher attachment strength than the western.

In each trail, individuals from the eastern lineage always gaped significantly more than those from the western lineage (two-way ANOVA, square-root transformed for the first trial only, p < 0.001; Figure 2B). *Post-hoc* SNK tests indicated that both lineages gaped significantly more at hour one than at hours two or three during the first trial (p < 0.001), however, during the second trail, there was a time × lineage interaction (p < 0.05) because at hour two eastern lineage individuals gaped more that at hour three.

*P. perna* in the western range reached a significantly higher intertidal elevation than on the east coast (three-way ANOVA, p < 0.001; n = 6; Figure 2C). However, mussels in the western and eastern ranges displayed the same lower vertical limit.

## Discussion

The microsatellite data revealed strong genetic division between the two geographically defined groups of populations, supporting previous findings based on mitochondrial and nuclear markers [18,19]. Populations from cluster one inhabit the warm-temperate, south-western coast of South Africa, while cluster two comprises populations distributed along the sub-tropical eastern shores. Most importantly, genetic admixture was only moderate, asymmetric and not restricted to the previously described 200 km contact zone, which lies mostly in the warm-temperate transition zone [18]).

The admixture detected with microsatellite markers is probably the result of secondary contact between ancient lineages; phylogeographic patterns based on mtDNA (COI) and nDNA (ITS) sequence data indicate an Indo-Pacific origin of the species and a dispersal into the Mediterranean and Atlantic through the Tethys seaway. After the closing of the seaway, the two non-sister lineages diverged (310 kyr) and came into secondary contact after their respective eastern, or western, southward expansion [19]. However, genetic differentiation between lineages can be primarily attributed to the magnitude of the differences in allele frequencies rather than to unique alleles, as would be expected in ancient isolated lineages.

The detected gene exchange was primarily unidirectional, from east to west. Additionally, the sampled location at the geographic border of the two lineages (PA) shows the strongest gene intermediacy. Gene exchange and the maintenance of this pattern could be attributed to several non-mutually exclusive effects. Eastward gene flow could be impeded by dispersal barriers (e.g. currents), or by selection (i.e. unfavourable environmental conditions) causing outbreeding depression if lineages are locally adapted. Alternatively, poor mating success between lineages could also play a role, either due to divergent reproductive ecology, or to some level of genetic incompatibility.

### Barriers to dispersal

Mussel larval dispersal is largely influenced by local hydrodynamics. The main oceanographic influence on the east and south coasts of South Africa is the Agulhas Current. This is a major ocean current that flows towards the southeast, bringing warm, oligotrophic water from the Mozambique channel [52]. The inshore thermal front of this current varies geographically and can bring its warm waters very close inshore [53,54]. From near PA southwards the current is deflected away from the coast. The trajectories of nearshore drogues released off the south coast revealed no mixing of southern waters into the eastern water mass, while drifters released off the east coast moved southward, caught up in the Agulhas Current [20]. Although there are wind-driven surface currents that could allow limited eastward larval dispersal [55], water exchange is predominantly unidirectional, promoting westerly larval dispersal of the eastern lineage and explaining the asymmetrical gene flow described here. Previous mtDNA data on several species with planktonic larvae (including *P. perna*; [56]) support our results obtained with microsatellite data, by showing asymmetric east-to-west migration along the east coast (but see [8]).

Interestingly, one of our most eastern locations (DU) displayed a fair amount of genetic admixture. DU is the largest and busiest shipping terminal on the African Continent, and globally one of the seven key epicentres for inter-regional exchange of species [57]. Shipping activities are known vectors of larval dispersal [58,59]. The fact that DU is the only location on the east coast with these characteristics suggests that the introduction of non-indigenous genes from the western range detected only here is a consequence of human-mediated larval transport.

### Departures from random mating

Out of the six populations sampled, five displayed significant heterozygote deficiency. Null alleles are unlikely to be the cause of high F_is_ values in this case, since microsatellite loci with a high frequency of null alleles were excluded from the analyses, while the remaining loci compiled in the final dataset displayed similar homozygote excess simultaneously across all loci. Inbreeding is also unlikely in a broadcast spawner with long a larval pelagic phase (assumed to be similar to that of Mytilus spp., [60,61]) as recruitment is expected to produce spatially random kinship.

One possible explanation for heterozygote deficiency is a Wahlund effect due to the presence of genetically distinct subpopulations (which may be in HW equilibrium) in each population sampled. In this context, it is interesting that PA, which is at the contact zone between the two lineages, displayed the lowest mean observed heterozygosity, although this was not true across all loci (see Additional file 3: Table A1). Such effects can be expected when pooling discrete subpopulations with different allele frequencies that do not interbreed as a single randomly mating unit. In contrast, non-significant levels of heterozygote deficiency were detected in PO, which is the farthest population from the contact zone and located on the east side of the coast.

Reproductive isolation of the lineages, leading to heterozygote deficiency, could result from differences in the timing and frequency of spawning events [46,62,63]. It is not known when each lineage spawns nor what triggers the event, but lack of synchrony in spawning by co-existing mussel species has been reported, and analogous intraspecific differences may occur between lineages of *P. perna* [46].

### Selection

Behaviour can radically moderate an organism’s experience of the environment and so dictate physiological reactions, and consequently, tolerance limits to the stress imposed by those environmental conditions. We show that the two *P. perna* mtDNA lineages exhibit clear differences in behavioural traits, indicating an intraspecific behavioural divergence that potentially influences vertical zonation and habitat segregation over large and small spatial scales. By acclimating and comparing individuals from different locations in a common laboratory environment (common garden) we could differentiate between adaptive and plastic responses to distinct environmental conditions. The maintenance of behavioural phenotypic divergence in experimental populations indicated that differences are due to underlying genetic divergence. Mussel gaping, the intermittent opening and closure of the valves, allows the maintenance of aerobic respiration by sustaining an oxygen gradient across the mantle wall and the gills during emersion. Respiration rates of organisms usually increase with temperature and several studies have shown that oxygen consumption by mussels increases exponentially with temperature (e.g. [64]). In *P. perna*, gaping significantly increased with temperature, supporting the important role of this behaviour in aerobic respiration [65] and the correlation between gaping and heat stress.

Although gaping has advantageous respiratory effects, it results in water loss caused by both evaporation and incidental discharge of water during valve closure, thus increasing the risk of desiccation [47]. Although valve opening suggests possible evaporative cooling, several studies have excluded any link between this behaviour and the body temperature of isolated individuals [66,67] (but see [68] for emergent cooling effects in mussel aggregations).

The adaptive significance of gaping behaviour helps explain the vertical intertidal zonation of the western lineage of *P. perna* and the coexisting invasive *Mytilus galloprovincialis,* a non-gaping mussel species [47,68] highlighting how even very simple behaviour can influence the outcome of interactions between coexisting species.

Our results and previous studies [47,68] suggest a determinant effect of gaping on the small and large scale spatial distributions of *P. perna* lineages. More frequent gaping in the eastern lineage would promote efficient respiration under high, subtropical air temperatures, but more gaping would simultaneously increase the risk of desiccation, restricting the eastern lineage to lower on the shore than western conspecifics ([as has been shown for a comparison of *P*. *perna* with *M*. *galloprovincialis*; [47]).

In addition to differences in gaping rates, the eastern lineage showed much stronger attachment to the substratum. Firm byssal attachment is a prerequisite for survival in the intertidal (e.g. [69,70]) but this difference is unlikely to explain large scale spatial segregation of the lineages as the temperate and subtropical regions are not known to differ significantly in terms of wave action.

Temperature offers a more likely explanation as it has direct or indirect effects on attachment. Higher temperatures promote byssal thread production while weakening attachment by accelerating thread deterioration and negatively affecting the curing and moulding of byssal threads [71,72]. Additionally, collagenases are common in marine bacteria [73] and their activity is enhanced by temperature [74], potentially contributing to the weakening of the byssus. Thus, the greater attachment strength of mussels from the east coast could be an adaptive response to the thermally more challenging subtropical environment.

## Conclusions

This study shows how distinct genetic lineages, partially segregated along a sharp geographic environmental gradient display adaptive responses to different selective pressures. *P. perna* inhabits shores that are experiencing rapid and geographically uneven environmental changes [75,76]. Our results clearly indicate that, rather than the species exhibiting a common overall response, the two lineages are likely to be affected by, and react quite differently to, climate change. Our findings highlight how critical it is to understand intraspecific diversity if we wish to have a grasp of the real magnitude of biodiversity and how within-species diversity will shape ecosystem resilience/resistance in a changing environment.

### Availability of supporting data

The data set supporting the results of this article is included within the article (Additional file 5: Table A2).

## Additional files

Additional file 1: Figure A1. Frequencies of null alleles per locus per population. Estimates based on the algorithm presented in Brookfield [24] by MICROCHECKER. Additional file 2: Figure A2. Allele frequencies of each location. Each box represents one locus, according to the following order: P01, P05, P11, P27, P29, P02, P08, P16, P20, P26. Additional file 3: Table A1. Observed heterozygosity calculated for each locations and locus. Estimates made in GENETIX program [25]. Additional file 4: Figure A3. Identifying the best number of K cluster. Plot of mean posterior probability (LnP(K)) values (open quadrats) per cluster (K) based on 20 replicates per K, generated by the STRUCTURE program [32] and delta K analysis (filled circles) of LnP(K) [33]. Additional file 5: Table A2. Genotyping Table. Scored alleles per location and per locus.
