# Supplementary figures and images for: Intraspecific genetic lineages of a marine mussel show behavioural divergence and spatial segregation over a tropical/subtropical biogeographic transition
Source: BMC Evol Biol. 2015 May 31;15:100. doi: 10.1186/s12862-015-0366-5 (PMC4449970; doi:10.1186/s12862-015-0366-5)

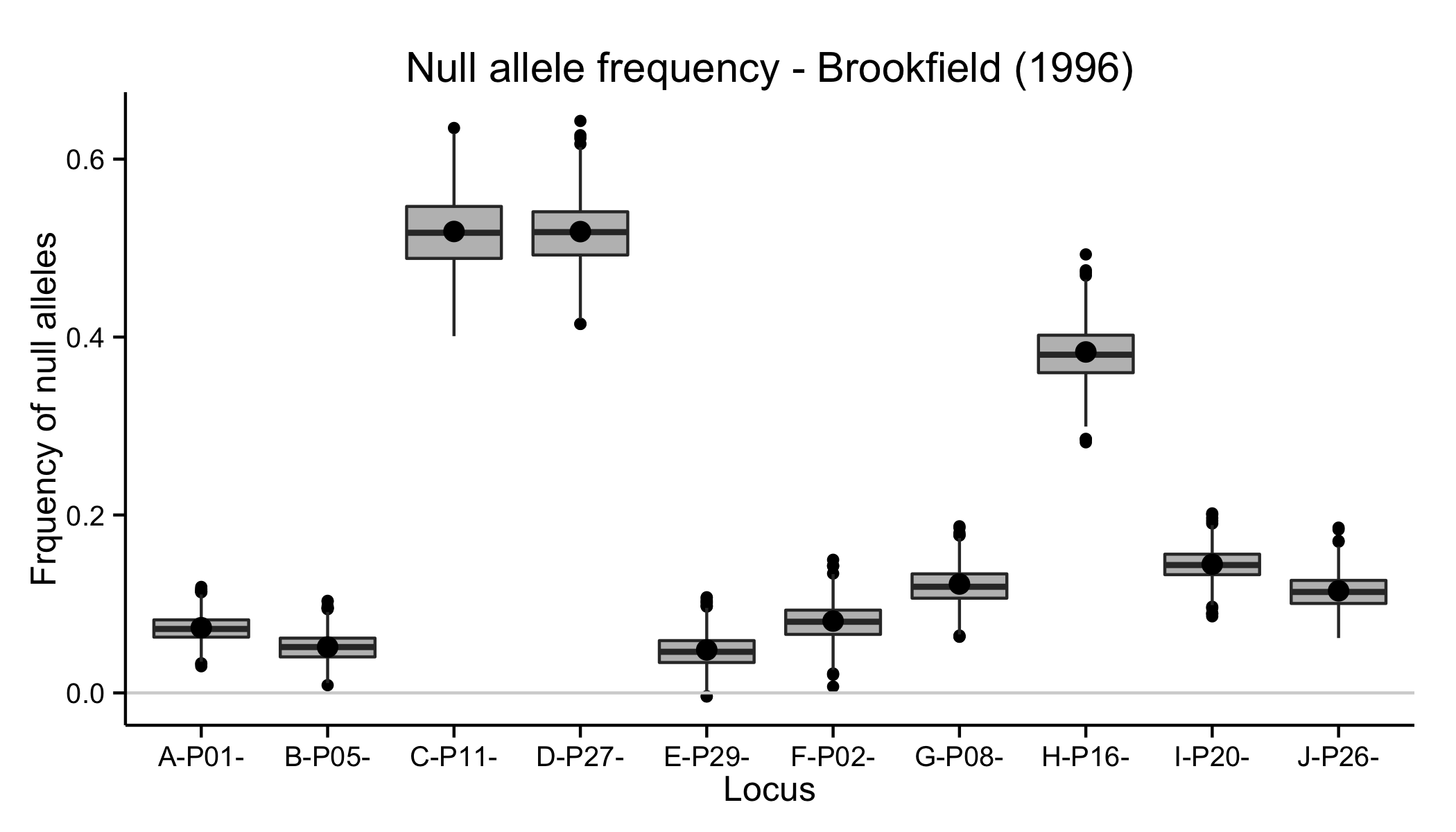

Supplement: Additional file 1: Figure A1. — Frequencies of null alleles per locus per population. Estimates based on the algorithm presented in Brookfield [24] by MICROCHECKER. [file 12862_2015_366_MOESM1_ESM.png]

P01

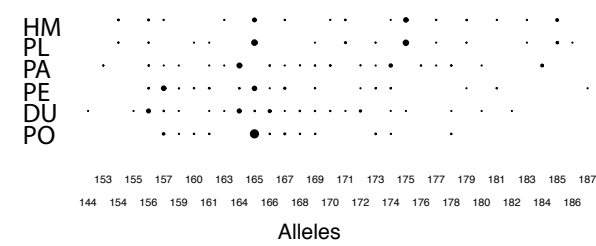

P05

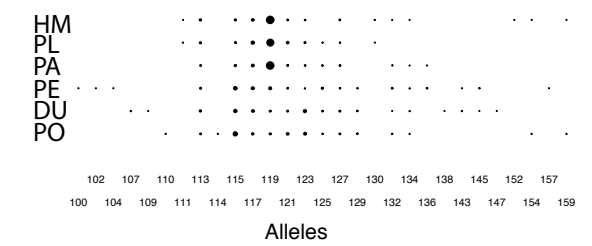

P11

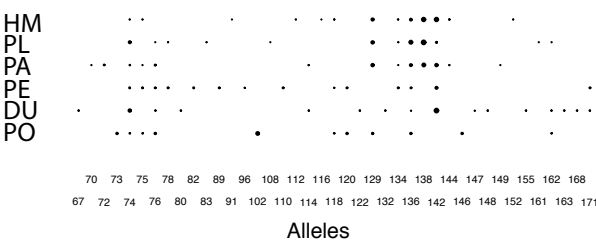

P27

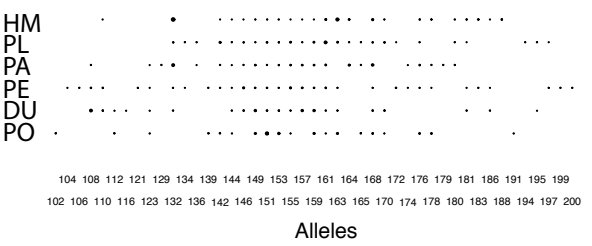

P29

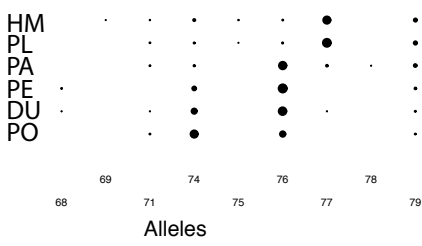

P02

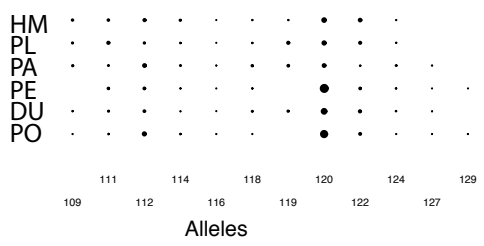

P08

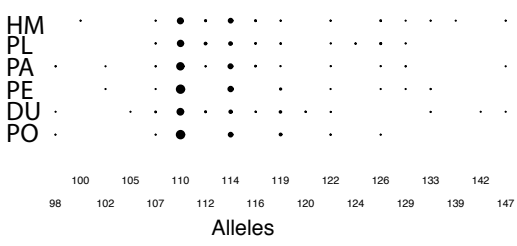

P16

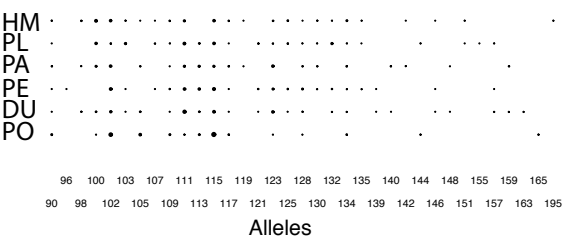

P20

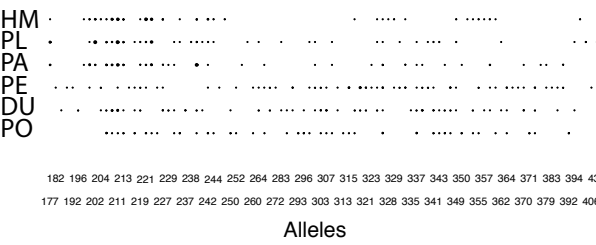

P26

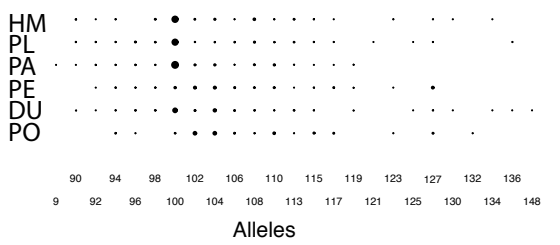

Supplement: Additional file 2: Figure A2. — Allele frequencies of each location. Each box represents one locus, according to the following order: P01, P05, P11, P27, P29, P02, P08, P16, P20, P26. [file 12862_2015_366_MOESM2_ESM.pdf]

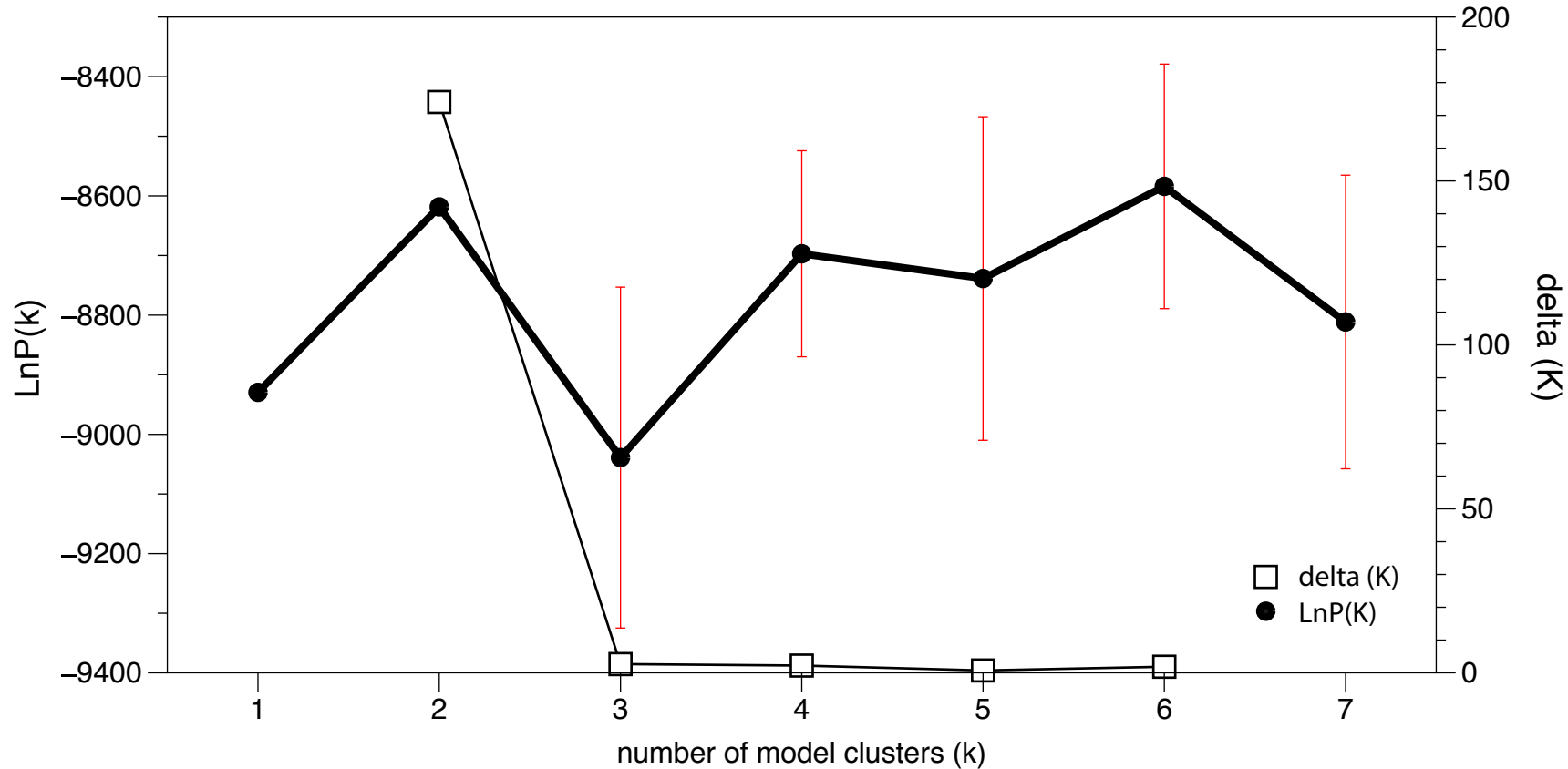

Supplement: Additional file 4: Figure A3. — Identifying the best number of K cluster. Plot of mean posterior probability (LnP(K)) values (open quadrats) per cluster (K) based on 20 replicates per K, generated by the STRUCTURE program [32] and delta K analysis (filled circles) of LnP(K) [33]. [file 12862_2015_366_MOESM4_ESM.pdf]
