# Supplementary material for: Intraspecific genetic lineages of a marine mussel show behavioural divergence and spatial segregation over a tropical/subtropical biogeographic transition
Source: BMC Evol Biol. 2015 May 31;15:100. doi: 10.1186/s12862-015-0366-5 (PMC4449970; doi:10.1186/s12862-015-0366-5)
Supplement: Additional file 3: Table A1. — Observed heterozygosity calculated for each locations and locus. Estimates made in GENETIX program [25]. [file 12862_2015_366_MOESM3_ESM.docx]

|  | HM | PL | PA | PE | DU | PD |
| --- | --- | --- | --- | --- | --- | --- |
| P01 | 0.875 | 0.7609 | 0.7778 | 0.8667 | 0.7447 | 0.6296 |
| P05 | 0.7234 | 0.7273 | 0.6512 | 0.8667 | 0.7857 | 0.9 |
| P29 | 0.7391 | 0.6591 | 0.5152 | 0.6471 | 0.7778 | 0.875 |
| P02 | 0.6667 | 0.4583 | 0.6889 | 0.8696 | 0.75 | 0.7931 |
| P08 | 0.6667 | 0.6458 | 0.4468 | 0.4103 | 0.587 | 0.5556 |
| P20 | 0.617 | 0.7083 | 0.7442 | 0.8 | 0.6957 | 0.8333 |
| P26 | 0.7083 | 0.7917 | 0.6364 | 0.7174 | 0.6875 | 0.7241 |
|  |  |  |  |  |  |  |
| Mean | 0.714 | 0.679 | 0.637 | 0.74 | 0.18 | 0.759 |
